# An examination of the relationship between negative emotions and family dynamics in individuals with internet addiction

**DOI:** 10.3389/fpsyt.2025.1628723

**Published:** 2025-07-08

**Authors:** Yao Chen, Xiwen Tian, Xuhui Zhou

**Affiliations:** ^1^ The School of Clinical Medicine, Hunan University of Chinese Medicine, Changsha, Hunan, China; ^2^ Department of Addiction Medicine, Hunan Institute of Mental Health, The Second People’s Hospital of Hunan Province (Brain Hospital of Hunan Province), Changsha, Hunan, China

**Keywords:** internet addiction, depression, anxiety, family intimacy, family adaptability

## Abstract

**Background:**

This study investigates the characteristics and typologies of family cohesion and adaptability among individuals diagnosed with Internet Addiction Disorder (IAD), and explores the associations between these family dynamics and levels of depression and anxiety. The findings aim to inform the theoretical underpinnings of family-based therapeutic interventions for IAD.

**Methods:**

A cross-sectional survey design was adopted, employing four well-validated instruments: the Young Diagnostic Questionnaire for Addiction, the Family Cohesion and Adaptability Scale, the Depression Scale, and the Anxiety Scale. The sample comprised 150 individuals diagnosed with IAD from the outpatient department of the Second People’s Hospital of Hunan Province, and a control group of 150 age- and gender-matched individuals without IAD. Statistical analyses, including independent sample t-tests and Pearson correlation analyses, were conducted using SPSS version 27.0.

**Results:**

(1) Compared to the control group, individuals with IAD reported significantly lower scores in both actual intimacy and actual adaptability, as well as in ideal adaptability, on the FACES scale (all P < 0.001). Dissatisfaction scores for both intimacy and adaptability were significantly higher in the IAD group (P < 0.002). The predominant family typologies identified in the IAD group were disengaged (49.3%) and rigid (80.0%). (2) Levels of depression and anxiety were significantly elevated in the IAD group compared to the control group (P < 0.001). (3) Actual and ideal scores of family intimacy and adaptability were negatively correlated with depression and anxiety scores in the IAD group (P < 0.05).

**Conclusion:**

The findings highlight the necessity of a dual-focused intervention strategy. Preventive programs should prioritize individuals exhibiting lower levels of family cohesion and adaptability, with an emphasis on educating parents about the crucial influence of family dynamics on child development and providing guidance on improving the home environment. Concurrently, attention to the mental health of children is essential. Early identification and intervention for symptoms of depression and anxiety may help prevent the escalation of comorbid conditions such as Internet Addiction Disorder and emotional dysregulation, thereby enhancing the effectiveness of therapeutic outcomes.

## 1 Introduction

Internet addiction (IA) is characterized by excessive and uncontrolled internet use, leading to significant psychological distress, social difficulties, and functional impairments (1). Sharing similarities with other behavioral addictions, IA has been associated with adverse outcomes in interpersonal relationships, family functioning, and broader social domains. The global prevalence of IA has been steadily increasing, particularly among adolescents and young adults (2). In China, the issue is especially pronounced, with recent estimates indicating a prevalence rate of approximately 5.19% among students (3).

During adolescence, emotional regulation capacity remains underdeveloped, rendering young individuals more susceptible to mental health problems associated with Internet Addiction (IA) (4).These issues include attention-deficit/hyperactivity disorder (ADHD) (5), anxiety disorders, depression (6), stress, low self-esteem, social anxiety, and overall impaired psychological well-being (7). Therefore, maintaining appropriate internet use is essential for minimizing potential harm among children and adolescents. According to the displacement theory, excessive internet use may reduce intimacy within family relationships and limit social engagement, which in turn can strain interpersonal connections and exacerbate psychological distress (8).

The home environment plays a critical role in shaping adolescents’ social interactions and behavioral development. *Family cohesion* refers to the emotional bonds among family members, whereas *family adaptability* denotes the family’s capacity to adjust its power structure, roles, and relationships in response to developmental challenges or situational demands. Previous research suggests that a balanced level of cohesion and adaptability is most conducive to healthy family functioning, while extreme levels—either too low or too high—are associated with dysfunctional family dynamics (9). Empirical studies have demonstrated significant negative correlations between family intimacy and adaptability and the risk of Internet Addiction (IA). Specifically, low levels of family cohesion and poor adaptability—characterized by frequent family conflicts and harsh parental discipline—have been identified as contributing factors to IA behaviors. In contrast, a secure and supportive family environment has been shown to enhance adolescents’ productivity, subjective well-being, and life satisfaction, while reducing the likelihood of maladaptive behaviors (10). Moreover, family cohesion also appears to influence emotional functioning. Negative emotions, such as depression, anxiety, and stress, often emerge as psychological responses to adverse familial environments and are recognized as important predictors of IA (11). Adolescents experiencing low family intimacy are more vulnerable to negative emotional states, which in turn significantly increase the risk of developing IA (12).

This study aims to examine the associations between negative emotions, family cohesion, and adaptability in individuals with Internet Addiction (IA), with the objective of informing more effective interventions and improving clinical outcomes in IA treatment.

## 2 Methods

### 2.1 Design

This study utilized a mixed-methods approach incorporating both questionnaire-based surveys and clinical interviews. The Young Diagnostic Questionnaire for Internet Addiction and relevant exclusionary diagnoses were administered by qualified psychiatrists. Participants completed a set of standardized self-report instruments, including a general demographic questionnaire, the Family Adaptability and Cohesion Evaluation Scales (FACES), and validated measures of depression and anxiety.

### 2.2 Participants

The Internet Addiction (IA) group consisted of 150 male patients diagnosed with IA who received outpatient or inpatient treatment at the Addiction Medicine Center of the Second People’s Hospital of Hunan Province between July 1, 2024, and March 1, 2025.

Inclusion criteria for the IA group were as follows:

(a) A score meeting diagnostic criteria for IA on Young’s Diagnostic Questionnaire, jointly evaluated by a psychiatrist, the adolescent, and their parents;
(b) Internet use resulting in academic withdrawal, suspension, significant academic decline, inability to maintain employment, or marked impairment in family and social relationships;
(c) Symptom duration and severity consistent with IA for at least three months.

Exclusion criteria included:

(a) Co-occurring psychiatric disorders, including neurosis, mood disorders, schizophrenia, conduct disorder, personality disorders, and attention-deficit/hyperactivity disorder (ADHD), as well as current use of psychotropic medications such as antidepressants or anxiolytics;
(b) A history of severe childhood trauma (e.g., abuse, major loss) or current residence in single-parent or separated families, based on Family Assessment Device (FAD) scores indicating severe family dysfunction.

The control group comprised 150 students, matched to the IA group by age and gender through stratified sampling. Participants were randomly selected from primary schools, middle schools, high schools, and vocational colleges in Changsha during the same study period.

Inclusion criteria for the control group were:

(a) A score <5 on Young’s Diagnostic Questionnaire, jointly assessed by a psychiatrist, the adolescent, and their parents;
(b) No significant negative impact of internet use on family or peer relationships, academic/work performance, or behavioral violations (e.g., truancy, fighting, or legal infractions);
(c) No history of mental or substance use disorders;
(d) Basic proficiency in computer operation.

### 2.3 Ethical considerations

This study was approved by the Medical Ethics Committee of the Medical College of Shenzhen University (Approval No. PN-202400066). All participants were fully informed of the study’s purpose and procedures prior to participation. Written informed consent was obtained from all participants before the administration of any questionnaires.

### 2.4 Tools

#### 2.4.1 Demographic characteristics

A self-designed questionnaire was used to collect general sociodemographic information. The items covered participants’ gender, age, grade level, place of residence, only-child status, parental educational levels, and other relevant demographic variables. In addition, questions related to patterns of internet usage were included. Young’s Diagnostic Questionnaire for Internet Addiction.

The Young’s 8-item Diagnostic Questionnaire for Internet Addiction was employed (13). Each affirmative response was scored as 1 point, while a negative response was scored as 0 points. Participants with a total score of ≥5 were classified as having Internet Addiction (IA). To minimize the risk of response bias or intentional denial by adolescents with IA, the questionnaire was administered by psychiatrists or psychologists, who conducted simultaneous interviews and assessments with both the patients and their parents. In the present study, the scale demonstrated excellent internal consistency, with a Cronbach’s alpha coefficient of 0.93. Family Adaptability and Cohesion Evaluation Scales.

The Chinese version of the Family Adaptability and Cohesion Evaluation Scales (FACES) (14) was utilized to assess family cohesion and adaptability. This instrument has demonstrated good reliability and validity in Chinese populations (15). The scale comprises two dimensions: cohesion and adaptability, encompassing a total of 30 items. Each item is rated on a 5-point Likert scale (1 = never, 2 = rarely, 3 = sometimes, 4 = often, 5 = always), reflecting the frequency with which the described situation occurs within the family. Participants responded to each item twice: once to describe their actual perceptions of their current family environment, and once to indicate their ideal family environment. Scores for actual and ideal family functioning were calculated separately, with higher scores indicating greater family cohesion and better adaptability. Family cohesion is categorized into four types ranging from low to high: disengaged, connected, cohesive, and enmeshed. Family adaptability is similarly categorized into four types from low to high: rigid, structured, flexible, and chaotic. The Cronbach’s alpha coefficient for this scale in the present study was 0.89.

#### 2.4.2 Self-Rating Depression Scale

The Self-Rating Depression Scale (SDS), developed by Professor Zung at Duke University in 1965 (16), was employed to assess the severity of depressive symptoms in the study population. Its reliability and validity have been established in Chinese populations (17). The scale consists of 20 items, with respondents rating each item on a 4-point scale (1 to 4) according to their subjective experiences over the past week. The scale includes an equal number of positively and negatively worded items (10 each). The raw score is calculated by summing all item scores, and the standard score is derived by multiplying the raw score by 1.25. In this study, the Cronbach’s alpha coefficient was 0.79.

#### 2.4.3 Self-Rating Anxiety Scale

The Self-Rating Anxiety Scale (SAS), developed in 1971 by Professor Zung, a Chinese-American scholar at Duke University (18), was utilized to evaluate the subjective anxiety levels of participants. The Chinese version of the scale has been widely applied and demonstrates adequate reliability and validity (19) The scale comprises 20 items, each rated by respondents on a 4-point Likert scale (1 to 4), reflecting their feelings over the past week. The scale includes 15 positively worded items and 5 negatively worded items. The raw score is calculated by summing the scores of all items, and the standard score is derived by multiplying the raw score by 1.25. In this study, the Cronbach’s alpha coefficient was 0.81.

## 3 Statistical analysis methods

Statistical analyses were conducted using SPSS version 27.0. Independent samples t-tests were applied to compare continuous variables between groups, while Pearson correlation analysis was employed to assess relationships between variables. Categorical data were analyzed using chi-square (χ²) tests. Prior to performing independent samples t-tests, the data were examined for normality using the Shapiro-Wilk test and for homogeneity of variances using Levene’s test. If the assumptions of normality (P > 0.05) and homogeneity of variance (P > 0.05) were satisfied, independent samples t-tests were used; otherwise, the non-parametric Mann-Whitney U test was applied. A significance threshold of α = 0.05 was adopted.

## 4 Results

### 4.1 Comparison of sociodemographic characteristics between groups

The comparison of basic sociodemographic characteristics between the Internet Addiction (IA) group and the non-IA control group (**Table 1**) showed no statistically significant differences in gender, age, grade, or living arrangements with parents (P = 0.348–0.963). However, the IA group had a significantly higher proportion of individuals residing in urban areas, attending school as day students, and being only children, compared to the control group (P < 0.05).

**Table 1:** Analysis of basic characteristics and factors related to internet addiction in two groups (including effect size).

Variables	IA Group (n=150)	Control Group (n=150)	t/χ2	P	Cohen’s *d/Cramér’s V*
Age (x ± s, a)	15.80± s2.23	15.75 ± 2.17	t=0.231	0.817	
10–15 years old	69 (46.0%)	67 (44.7%)			
16–20 years old	81 (54.0%)	83 (55.3%)			
Gender: Male/Female	98/52	92/58	χ2 = 0.517	0.472	
Grade:			χ2 = 0.282	0.963	
Primary School	4 (2.7%)	3 (2.0%)			
Junior High School	52 (34.7%)	52 (34.7%)			
Senior High School	65 (43.3%)	68 (45.3%)			
University	29 (19.3%)	27 (18.0%)			
Residence			χ 2 = 9.794	0.002	Cramér’s V=0.18
City	95	68			
Small town or rural-urban fringe/Rural	55	82			
Student Type			χ2 = 6.454	0.011	Cramér’s V=0.15
Day student	85	63			
Boarding student	65	87			
Only child: Yes/No	54/96	21/129	χ2 = 19.360	<0.001	Cramér’s V=0.25
Living with parents: Yes/No	136/14	126/24	χ2 = 3.447	0.348	
Closeness					
Actual closeness (x ± s)	55.8± s11.45	66.9± s12.32	t =8.059	<0.001	Cohen’s *d=0.93*
Ideal closeness (x ± s)	66.3± s13.03	68.51± s12.32	t =1.452	0.148	
Closeness dissatisfaction (median, range)	10, 49	3, 41	z=-7.769	<0.001	Cohen’s *d=*0.94
Adaptability					
Actual adaptability (x ± s)	36.2± s10.50	46.8± s10.40	t =8.797	<0.001	Cohen’s *d=1.02*
Ideal adaptability (x ± s)	52.31± s11.33	47.57± s10.64	t =-3.735	<0.001	Cohen’s *d=0.43*
Adaptability dissatisfaction (median, range)	14, 51	3, 37	z=-11.087	<0.001	Cohen’s *d=1.46*
SDS score (x ± s)	62.98± s14.82	34.05± s26.90	t=-11.539	<0.001	Cohen’s *d=0.92*
SAS score (x ± s)	51.93± s15.89	28.89± s24.06	t=-9.789	<0.001	Cohen’s *d=0.56*

A Cohen’s d value greater than 0.8 indicates a large effect size, between 0.5 and 0.8 indicates a medium effect size, and less than 0.5 indicates a small effect size.

### 4.2 Comparison of Family Adaptability and Cohesion Evaluation Scale scores between groups

**Table 1** summarizes the basic characteristics of the two groups and the analysis of variables associated with Internet addiction. The IA group exhibited significantly lower scores in actual cohesion, as well as actual and ideal adaptability, compared to the control group (P < 0.001). Additionally, the IA group showed significantly higher dissatisfaction scores for both cohesion and adaptability (P < 0.001). These differences were statistically significant. In contrast, no significant difference was observed in ideal cohesion scores between the two groups (P = 0.148).

### 4.3 Comparison of depression and anxiety scores between groups

As shown in **Table 1**, the IA group exhibited significantly higher depression and anxiety scores compared to the control group, with these differences reaching statistical significance (P < 0.001).

### 4.4 Comparison of family cohesion and adaptability types between groups

**Table 2** summarizes the distribution of family cohesion and adaptability types between the two groups [n (%)]. Significant differences were found in the composition of both family cohesion and adaptability types, with all comparisons reaching statistical significance (P < 0.05). The IA group predominantly exhibited disengaged and separated family cohesion types, alongside primarily rigid family adaptability. In contrast, the control group displayed a relatively balanced distribution across all four family cohesion types, with family adaptability mainly characterized as structured and flexible.

**Table 2:** Comparison of family cohesion and adaptability type distribution between the two groups [n (%)].

Factor	IA Group (n=150)	Control Group (n=150)
Family intimacy		
Unconsolidated	74 (49.3)	25 (16.7)
Autonomous	35 (23.3)	33 (22.0)
Intimate	27 (18.1)	33 (22.0)
Enmeshed	14 (9.3)	59(39.3)
Family adaptability		
Rigid	120 (80.0)	27 (18.0)
Structured	15 (10.0)	61 (40.7)
Flexible	13(8.7)	41 (27.3)
Irregular	2 (1.3)	21 (14.0)

Comparison of the distribution of cohesion and adaptability types between the IA group and the control group; p < 0.05.

### 4.5 Correlation between family cohesion and adaptability scores and depression and anxiety scores

**Table 3** displays the correlation analysis results (r, 95% CI) between family cohesion and adaptability scores and negative emotion scores. In the IA group, actual cohesion, actual adaptability, and ideal adaptability scores were significantly negatively correlated with both depression and anxiety scores (P < 0.01). Additionally, a modest negative correlation was observed between ideal cohesion scores and anxiety scores (r = -0.191, P < 0.05).

**Table 3:** Correlation analysis between family cohesion and adaptability and negative affect scores (r, 95% CI).

Factor	SDS(r, 95% CI)	SAS(r,95%CI)
Actual Proximity	-0.433**(-0.554,-0.293)	-0.291**(-0.431,-0.137)
Practical adaptability	-0.391**(-0.518,-0.246)	-0.251**(-0.395,-0.095)
Ideal Affinity	-0.310**(-0.448,-0.158)	-0.191*(-0.341,-0.032)
Ideal Adaptability	-0.327**(-0.463,-0.176)	-0.242**(0.388,-0.085)
Level of Intimacy Dissatisfaction	0.134(-0.027,0.288)	0.104(-0.057,0.260)
Adaptive dissatisfaction level	0.066(-0.096,0.224)	0.015(-0.146,0.175)

*P < 0.05, **P < 0.01.

## 5 Discussion

Previous studies have shown a correlation between the onset of Internet addiction (IA) and lower levels of family functioning, particularly reduced family cohesion (20) Consistent with these findings, our study revealed that compared to the control group, individuals with IA exhibited significantly lower actual scores of family intimacy on the FACES scale, as well as lower actual and ideal adaptability scores. These results suggest that IA patients often experience poorer and more dysfunctional family environments. Additionally, the IA group reported higher dissatisfaction scores for both intimacy and adaptability, indicating considerable dissatisfaction with their family dynamics.

These findings align with numerous prior studies, which have consistently demonstrated that individuals with IA tend to have lower levels of family intimacy and adaptability compared to non-IA populations (20). Furthermore, young adults with IA are more likely to come from dysfunctional family backgrounds than their non-addicted peers (21). Our sociodemographic analysis also revealed that the IA group had a higher proportion of urban residents, day students, and only children. This may be related to greater internet accessibility in urban areas and potentially reduced emotional support in only-child families. Although gender and age were matched between groups, socioeconomic factors could still influence patterns of internet use, suggesting a need for further adjustment and control of these variables in future research.

Furthermore, a substantial body of research has demonstrated the comorbidity or significant correlation between Internet addiction (IA) and various forms of psychological distress, including stress, depression, and anxiety (22). Consistent with these findings, our study showed that the IA group exhibited significantly higher scores for depression and anxiety compared to the control group. This suggests that individuals with IA are not only more likely to experience reduced family intimacy and adaptability but also have an increased vulnerability to psychological disorders such as depression and anxiety.

Regarding the family structure of individuals with Internet Addiction (IA), this study found that the IA group was predominantly characterized by disengaged (loose) and rigid family types. It is hypothesized that family members within this group, especially parent-child dyads, experience limited emotional exchange. Parents often adopt strict disciplinary approaches, paying insufficient attention to and understanding each other’s inner feelings. A balanced family environment—marked by adequate cohesion and flexibility—serves as a protective factor, offering individuals essential resources for self-determination without fostering maladaptive social media use (23). In contrast, dysfunctional and highly disengaged family functioning fails to satisfy individuals’ fundamental emotional needs, potentially leading to maladaptive compensatory behaviors online, such as seeking warmth and emotional support through social media that is lacking in real-life family interactions. These findings thus suggest that social media may become a compensatory outlet for emotional support absent within the family environment (24).

Furthermore, regarding the relationship between family functioning and negative emotions in patients with Internet Addiction (IA), this study found that family cohesion and adaptability were negatively correlated with both anxiety and depressive symptoms. These findings align with previous research, which indicates that high family cohesion and adaptability contribute to alleviating anxiety and depressive symptoms (25) In families with high cohesion, members are better able to express their emotions openly and show mutual respect (26). Similarly, high family adaptability reflects a flexible family environment, where members maintain interdependence while preserving appropriate individual autonomy (27). Existing studies suggest that families influence an individual’s psychological state primarily through child-rearing practices, communication patterns, and conflict resolution between parents and children (28). Therefore, family cohesion and adaptability facilitate overcoming emotional difficulties by providing channels for emotional communication, psychological comfort, and social support (29).

Several limitations should be acknowledged when interpreting the findings of this study. First, the cross-sectional design restricts the ability to infer causal relationships between Internet Addiction (IA), family functioning, and negative emotional states such as depression and anxiety; thus, only associations can be established. Future research employing longitudinal or experimental designs is necessary to clarify causality. Second, this study did not account for potentially confounding variables such as socioeconomic status (e.g., household income, parental occupation) or the detailed composition of screen time (e.g., educational versus recreational use), which may influence the observed relationships. Controlling for these factors in future studies could help elucidate the underlying causal pathways. Third, reliance on self-report instruments such as the Self-Rating Depression Scale (SDS) and Self-Rating Anxiety Scale (SAS) may introduce response biases, including underreporting or overreporting of symptoms. Future research should incorporate multi-method assessments, including clinical interviews, peer or parental reports, and behavioral observations, to improve measurement accuracy. Additionally, incorporating evaluations of coping strategies using behavioral assessments and specialized psychometric tools may provide a more comprehensive understanding of the mechanisms linking family functioning and IA.

## 6 Conclusion

This study investigated the associations among Internet addiction (IA), family cohesion and adaptability, and negative emotional states such as depression and anxiety. The findings underscore the significant relationship between impaired family functioning and the presence of negative emotional symptoms in individuals with IA, suggesting that such individuals frequently experience dysfunctional family dynamics and weakened familial relationships. Moreover, children raised in environments with poor family functioning appear more vulnerable to developing psychological disorders, including depression and anxiety. These findings highlight the need for targeted preventive interventions focusing on individuals with low levels of family cohesion and adaptability. It is essential to raise parental awareness regarding the critical role of family functioning in children’s psychological development and to provide guidance on enhancing the family environment. In parallel, attention should be directed toward the mental health of children by implementing early identification and intervention strategies for depressive and anxiety symptoms. Such measures may help prevent the co-occurrence and progression of IA and emotional disturbances, thereby facilitating more effective treatment outcomes.

## Data Availability

The raw data supporting the conclusions of this article will be made available by the authors, without undue reservation.
